# Regulatory Authority Evaluation of the Effectiveness and Efficiency of the ZaZiBoNa Collaborative Medicines Registration Initiative: The Way Forward

**DOI:** 10.3389/fmed.2022.898743

**Published:** 2022-04-25

**Authors:** Tariro Sithole, Gugu Mahlangu, Stuart Walker, Sam Salek

**Affiliations:** ^1^School of Life and Medical Sciences, University of Hertfordshire, Hatfield, United Kingdom; ^2^Medicines Control Authority of Zimbabwe, Harare, Zimbabwe; ^3^Centre for Innovation in Regulatory Science, London, United Kingdom; ^4^Institute for Medicines Development, Cardiff, United Kingdom

**Keywords:** ZaZiBoNa, regulatory harmonisation, work sharing, effectiveness, efficiency

## Abstract

**Introduction:**

ZaZiBoNa, the work-sharing initiative in the Southern African Development Community (SADC) that has been in operation for 8 years has successfully assessed over 300 dossiers/applications, with an overall median time to recommendation of 12 months. All 16 SADC countries participate in the initiative as either active or non-active members. While the successes of ZaZiBoNa are evident, some challenges still exist. The aim of this study was to solicit the views of the participating authorities on the effectiveness and efficiency of the current operating model of the ZaZiBoNa initiative.

**Methods:**

Data were collected in 2021 using the Process, Effectiveness and Efficiency Rating (PEER) questionnaire developed by the authors. The questionnaire was completed by the focal person in each country and approved by the head of the authority.

**Results:**

ZaZiBoNa serves as a platform for work sharing, information exchange, capacity building and harmonisation of registration requirements. One of the benefits to regulators has been the improvement in the capacity to conduct assessments. Manufacturers have benefited from compiling one package (modules 2–5) for the initial submission as well as a single response package to the consolidated list of questions, which saves time and resources. Respondents were of the view that patients have benefited as the ZaZiBoNa has contributed to an improved availability and accessibility to quality-assured medicines. Some of the challenges identified were the inadequacy of resources and differences in time to the implementation of ZaZiBoNa recommendations by the individual countries. The establishment of a regional unit hosted in one of the member countries to enable centralised submission and coordination was identified as the best strategy to improve the effectiveness and efficiency of the initiative in the interim, with the long-term goal being the establishment of a regional medicines authority.

**Conclusion:**

The study identified the strengths of the ZaZiBoNa initiative as well as the opportunities for improvement. The recommendations made would further strengthen this initiative.

## Introduction

In October 2013, the inaugural meeting of the ZaZiBoNa collaborative medicines registration initiative was held in Windhoek, Namibia (1). Named using the first two letters of the four founding countries in the Southern African Development Community (SADC), namely Zambia, Zimbabwe, Botswana and Namibia (2), ZaZiBoNa was a vision of the Heads of Agencies of those countries, with the support of the World Health Organization (WHO) prequalification team and the Southern African Programme on Access to Medicines and Diagnostics (SAPAM) (1). The main objectives of the ZaZiBoNa initiative were “a reduced workload, reduction in timelines to registration, the development of mutual trust and confidence in regulatory collaboration and to provide a platform for training and collaboration in other regulatory fields” (1).

Prior to the launch of the initiative, the national medicines regulatory authorities in SADC operated in isolation, despite facing similar challenges such as large registration backlogs that resulted in long registration times, hindering access to critical medicines by their populations (3). Poor retention of human resources, and inadequate capacity to assess certain types of medicinal products were also common challenges faced by the countries, making a collaborative approach involving sharing of resources and expertise not only desirable but absolutely imperative. The four countries signed memoranda of understanding agreeing to participate in the initiative and agreed that this would be a requirement for other SADC countries wishing to join the initiative (1). Today, all 16 SADC countries participate in the ZaZiBoNa initiative, either as active members or non-active members depending on their capacity to conduct dossier assessments and good manufacturing practice (GMP) inspections (1, 4). ZaZiBoNa was absorbed into the SADC medicines registration harmonisation project in 2015 which, together with other regional economic communities in Africa, is overseen by the African Medicines Regulatory Harmonisation Initiative (AMRH) (5).

**FIGURE 1:**
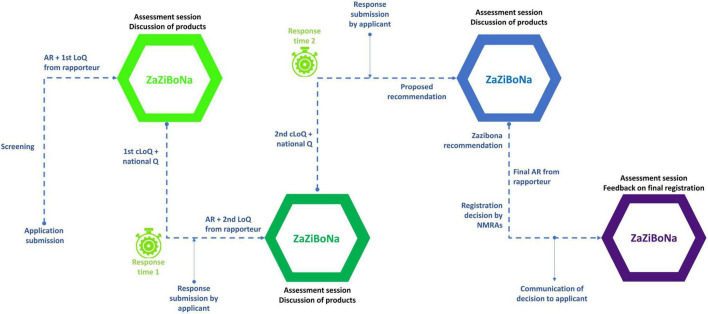
ZaZiBoNa process map. Reprinted from Sithole et al. (1) under a Creative Commons Attribution 4.0 International License. Copyright, the authors, 2020.

In the current model of the ZaZiBoNa initiative, applicants simultaneously submit applications for registration and pay fees to each of the countries in which they wish to market their medicinal product (1, 6, 7). The assessment of dossiers/applications is carried out using a rapporteur and co-rapporteur before consideration of the report by a group of assessors from all the active member countries. In the absence of a regional legal framework, ZaZiBoNa does not have centralised submissions or approvals/registrations (1). Therefore, once the evaluation is concluded, an assessment report with a recommendation and a consolidated list of questions is produced (1) and communication of the list of questions to the applicants as well as the final decision on the registration/marketing authorisation of medicinal products is left to the individual participating countries (1, 6). The process map is illustrated in Figure 1 (1). The Heads of Agencies serve as a governing body and countries participate in the initiative through multilateral agreements.

A key success of ZaZiBoNa has been its ability to continue operating with limited resources, with participating countries also contributing financially to the initiative since its inception (1). Another important initiative accomplishment is the achievement of shorter timelines for the 333 dossiers/applications that have been assessed to date (December 2021) compared with the timelines achieved by some of the participating countries using their national procedures (6, 8). For example, ZaZiBoNa has an overall median time to recommendation of 12 months (9), whereas some of the participating countries had approval times of over 650 days in 2020 (10). The gap in regulatory capacity among participating countries has also been reduced through the training of assessors and inspectors, bringing further harmonisation in the way assessments and GMP inspections are conducted in the SADC region.

Despite these successes, some challenges have been identified through feedback from applicants such as differences in time to implement ZaZiBoNa recommendations by the participating countries (1, 4). This is not surprising, as the participating countries have some differences in their registration processes; for example, frequency of expert Committee meetings (4, 11), which may affect the implementation of the ZaZiBoNa recommendations.

Sithole and colleagues therefore recommended a review of the ZaZiBoNa operating model to identify opportunities for improved efficiency (4). The aim of this study was to solicit the views of the authorities on the effectiveness and efficiency of the current operating model of the ZaZiBoNa initiative. To our knowledge, no similar study has been conducted or published in the literature.

## Study Objectives

### The Study Objectives Were to

1. Obtain the views of the individual medicines’ regulatory authorities of the ZaZiBoNa work-sharing initiative
2. Identify the challenges experienced by individual authorities since the inception of the ZaZiBoNa initiative
3. Determine the strengths and weaknesses of the initiative
4. Identify the ways of improving the performance of the initiative
5. Envisage the strategy for moving forward

## Materials and Methods

### Study Participants

All nine active members of the ZaZiBoNa initiative participated in the study translating to a response rate of 100%. These are, Botswana, Democratic Republic of Congo, Malawi, Mozambique, Namibia, South Africa, Tanzania, Zambia and Zimbabwe. Active member status is determined by “the capacity to conduct assessments and GMP inspections” (4).

### Data Collection

Data were collected in August 2021 using the Process, Effectiveness and Efficiency Rating questionnaire (PEER) developed by the authors. The questionnaire was completed by the focal person in each country and approved by the head of the authority. The questionnaire comprised five sections under the headings Demographics; Benefits of the ZaZiBoNa initiative; Challenges of the ZaZiBoNa initiative; Improving the performance (effectiveness and efficiency) of the work-sharing programme; and Envisaging the strategy for moving forward.

To examine the applicability and practicality of the PEER questionnaire, it was piloted with two member authorities in July 2021 prior to undertaking the main study. Subsequently, to establish the content validity and relevance of the PEER questionnaire, an additional questionnaire was completed and semi-structured interviews were carried out in September 2021 with each of the member authorities following completion of the questionnaire.

### Ethics Committee Approval

The study was approved by the Health, Science, Engineering and Technology ECDA, University of Hertfordshire, United Kingdom [Reference Protocol number: LMS/PGR/UH/04350].

## Results

For the purpose of clarity, the results are presented in five parts, matching the sections of the questionnaire: Part I—Demographics and authority resources; Part II—Benefits of the ZaZiBoNa initiative; Part III—Challenges of the ZaZiBoNa initiative; Part IV—Improving the performance of the work-sharing programme; and Part V—Envisaging the strategy for moving forward.

### Part I—Demographics and Authority Resources

The study respondents’ age ranged from 31 to 49 years, with a range of regulatory experience from 4 to 16 years. Five of the respondents were female and 4 were male. Authority resources, including the number of authority assessors assigned to ZaZiBoNa reviews are listed in Table 1.

**TABLE 1:** Authority resources.

	Botswana	D.R congo	Malawi	Mozambique	Namibia	South Africa	Tanzania	Zambia	Zimbabwe
Number of staff	93	300	53	87	17	237^#^	290^#^	120	135
Number of assessors*	9	75	8	9	8	33	59	18	20
Number of assessors involved in ZaZiBoNa	9	4	4	5	6	12	20	14	20

*Internal; ^#^Permanent.

### Part II—Benefits of the ZaZiBoNa Initiative

#### Benefits of the ZaZiBoNA Initiative

**FIGURE 2:**
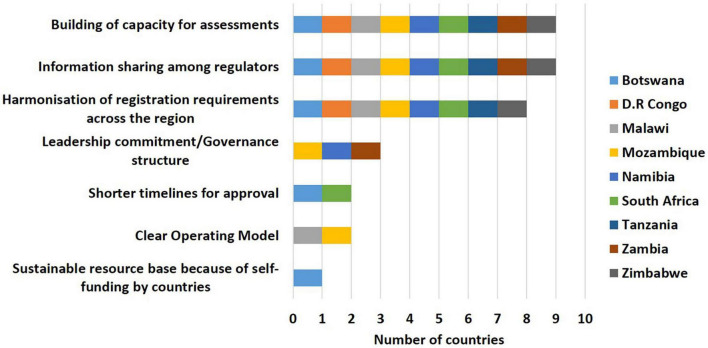
Benefits of the ZaZiBoNa initiative according to regulatory authority respondents.

Information sharing among regulators (9/9), building of capacity for assessments (9/9) and harmonisation of registration requirements across the region (8/9) were identified as the top 3 benefits of the ZaZiBoNa initiative by the countries. However, less than a third of the countries believed that assessment through ZaZiBoNa resulted in shorter timelines for approval of medicines (2/9) or that the operating model was clear (2/9) (Figure 2).

#### Strengths of the ZaZiBoNa Process at Country Level

The availability of information on the submission process and timelines for ZaZiBoNa dossiers/applications on the country website was selected as the top strength at a country level by most of the countries (6/9). The availability of a separate register and tracking, priority review and regular committee meetings, which enabled the timely recommendation of dossiers/applications were also identified as strengths by the majority of countries (5/9). However, less than one third of the countries (2/9) published a list of medicinal products approved under ZaZiBoNa on their website, which could be regarded as a weakness of the initiative

#### Benefits of the ZaZiBoNa Initiative to Member Countries (Regulators)

The majority of the countries agreed that the ZaZiBoNa initiative provided them with benefits that include training, which has improved the performance of the assessors (9/9), a platform for interaction and information exchange with other regulators (9/9), an improvement in the quality of dossiers submitted (8/9) and the ability to apply high standards of assessment regardless of the size of the country or maturity of regulatory authority (7/9). However, less than one third of the countries (2/9) believed that the sharing of the workload through ZaZiBoNa resulted in shorter timelines for approval than in the individual countries, confirming the observation that this is a weakness of the initiative.

#### Benefits of the ZaZiBoNa Initiative to Applicants

**FIGURE 3:**
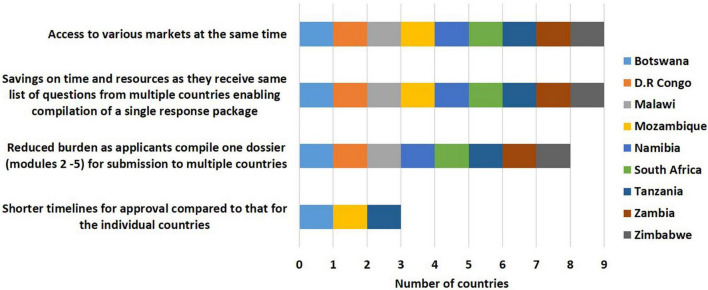
Benefits of the ZaZiBoNa initiative to applicants according to regulatory authority respondents.

Benefits to applicants selected by countries included reduction of the burden of compiling several dossiers for different countries, as only one dossier (modules 2–5) is required for submission to multiple countries through ZaZiBoNa (8/9) and savings in time and resources as the same list of questions is received from multiple countries enabling compilation of a single response package (9/9) with simultaneous access to various markets (9/9). However, only one third of the respondents (3/9) believed that applicants were receiving the promised benefit of shorter timelines for approval compared with timelines achieved for the individual countries (Figure 3).

#### Benefits of the ZaZiBoNa Initiative to Patients

Increased availability and access to quality-assured medicines (7/9) were identified as the benefits of the ZaZiBoNa initiative for patients by the majority of the countries, although that access was not regarded as always being faster than through individual countries (6/9). However, less than one third of the countries (2/9) were of the view that the initiative resulted in reduced prices of medicines.

### Part III—Challenges of the ZaZiBoNa Initiative

#### Challenges of the ZaZiBoNa Initiative

The top two challenges of the ZaZiBoNa initiative that were selected were the lack of centralised submission and tracking (8/9) and dependence on the member country processes for communication with applicants and expert committees (7/9). An unequal workload among member countries (5/9), lack of jurisdictional power (5/9), a low or decreasing number of applications (4/9) and lack of detailed information on the process for applicants (3/9) were also identified as challenges by the countries.

#### Challenges at a Country Level in Assessing ZaZiBoNa Dossiers/Applications

Inadequate human resources (8/9) and the failure by applicants to adhere to deadlines for response to questions (7/9) were cited as the greatest challenges at a country level. Additionally, the majority of the countries (5/9) were of the view that failure by manufacturers to follow the requirement to submit the exact same dossier to all countries of interest was an issue. The other challenges identified were poor record keeping and tracking (3/9), unpredictable scheduling of expert committee meetings (2/9), lack of buy-in from expert committees (1/9) and a failure by authorities to designate ZaZiBoNa assessments as part of the authority’s workload (1/9).

#### Challenges for Applicants Submitting Applications to the ZaZiBoNa Initiative

**FIGURE 4:**
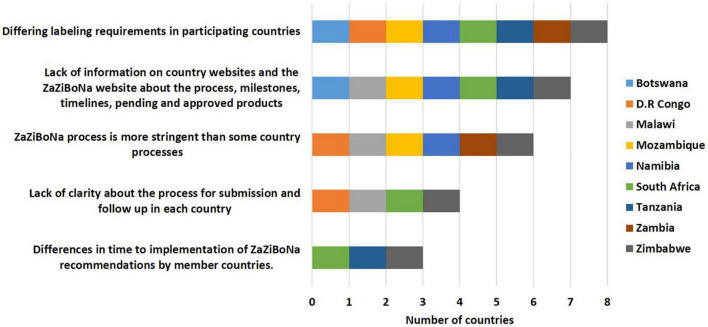
Challenges for applicants submitting applications to the ZaZiBoNa initiative according to regulatory authority respondents.

The majority of the countries agreed that differing labelling requirements in participating countries (8/9) and lack of information on individual country and ZaZiBoNa websites about the process, milestones, timelines and pending and approved medicinal products (7/9) were the greatest challenges faced by applicants with the initiative. Additionally, most of the countries were of the view that the ZaZiBoNa process is more stringent than some country processes (6/9), presenting a challenge for applicants. Other issues identified were lack of clarity about the process for submission and follow-up in each country (4/9) and differences in time to the implementation of ZaZiBoNa recommendations by member countries (3/9) (Figure 4).

### Part IV—Improving Performance (Effectiveness and Efficiency)

#### Ways to Improve the Effectiveness of the ZaZiBoNa Initiative

**FIGURE 5:**
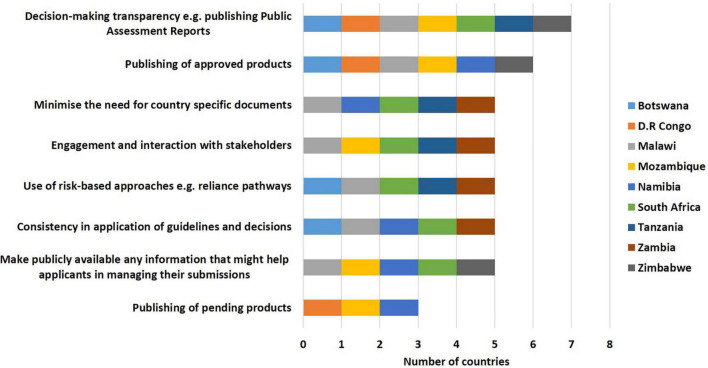
Ways to improve the effectiveness of the ZaZiBoNa initiative according to regulatory authority respondents.

Some of the ways identified by the countries to improve effectiveness of the initiative included decision-making transparency; for example, publishing public assessment reports (7/9), listing approved medicinal products (6/9), minimising the need for country-specific documents (5/9), engagement and interaction with stakeholders (5/9), use of risk-based approaches e.g., reliance pathways (5/9), consistency in application of guidelines and decisions (5/9), making information that might help applicants in managing their submissions publicly available (5/9) and publishing lists of pending dossiers/applications (3/9) (Figure 5).

#### Ways to Improve the Efficiency of the ZaZiBoNa Initiative

**FIGURE 6:**
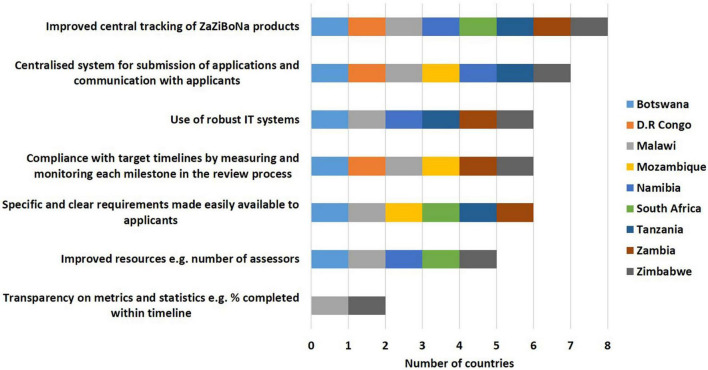
Ways to improve the efficiency of the ZaZiBoNa initiative according to regulatory authority respondents.

Improved central tracking of ZaZiBoNa dossiers/applications (8/9), a centralised system for submission of applications and communication with applicants (7/9), use of robust information technology systems (6/9), compliance with target timelines by measuring and monitoring each milestone in the review process (6/9), specific and clear requirements made easily available to applicants (6/9), improved resources; for example, number of assessors (5/9) and transparency on metrics and statistics; for example, percentage completed within timeline (2/9) were selected as ways to improve the efficiency of the initiative (Figure 6).

### Part V—Strategies for Moving Forward

The establishment of a regional unit hosted in one of the member states, to centrally receive and track ZaZiBoNa applications and be responsible for allocating work, apportioning the applicable fees to countries, tracking applications and communicating with applicants was selected by the majority of countries (8/9) as the best strategy moving forward in the interim. The majority of countries (7/9) were also of the view that to continue with the current operating model was the least effective strategy. All countries expressed the position that the establishment of a SADC regional medicines authority would be the best strategy, if it were legally possible, to address the challenges and areas requiring improvement in the initiative.

## Discussion

The results of this study show that the ZaZiBoNa initiative has achieved the majority of its objectives, which included facilitating greater information sharing and harmonisation of registration requirements. The capacity of countries to conduct assessments and inspections has markedly improved as a result of their participation in this initiative (4, 10). Reliance is being implemented within the initiative, as countries can quickly approve dossiers/applications that they have not reviewed but whose reports can be accessed through ZaZiBoNa. One of the key objectives of the ZaZiBoNa initiative was to reduce timelines for the approval of medicines, with a target median time of 9 months inclusive of the applicant’s time and the study results underscored the expected benefit to applicants of reduced timelines. However, the majority of countries did not believe that shorter timelines were being achieved and this may be problematic in the future, as it can negatively affect applicants’ interest and motivation to use this process. The additional challenges faced by applicants and acknowledged by the countries need to be addressed in order to make the initiative more attractive.

Clear communication of timelines for each milestone with applicants as well as the requirements for dossiers/applications to be reviewed will increase the applicants’ confidence in the process. At present, not all the participating countries have full information on ZaZiBoNa on their websites, including contact details of the focal person for follow-up. This is information that would be useful for applicants who may be planning submissions to ZaZiBoNa and is in place with other successful global work-sharing initiatives (8, 12). Some of the shortcomings at a country level can be attributed to inadequate resources, which may also impact the quality of the assessments. A weakness of this initiative that was identified from the study was the use of inexperienced assessors and the unavailability of experienced assessors in some of the countries to carry out the ZaZiBoNa work. The initiative should have standard operating procedures in place to ensure that only competent assessors and inspectors are seconded by the respective countries to participate in the initiative, an approach modelled on the Committee for Medicinal Products for Human Use (CHMP) of the European Medicines Agency (11).

It has been established that ZaZiBoNa uses an operating model similar to other global work-sharing initiatives (12–14); however, challenges have been identified. This could be due to the significantly lower resources; for example, the number of assessors, available to ZaZiBoNa countries when compared with countries in the other initiatives. Most of the active member countries in ZaZiBoNa are faced with the challenge of limited resources and a high number of applications (4, 10, 15–17) for the national procedure, which negatively impacts the work-sharing initiative. The use of a regional unit to coordinate assessments would also assist in addressing the identified challenges, particularly in a resource-constrained setting. In the long term, the establishment of a SADC regional medicines authority would be preferable and would address the challenge of the lack of jurisdictional power identified in this study.

Key recommendations to improve the effectiveness and efficiency of the ZaZiBoNa work-sharing initiative include:

• **Measuring and monitoring regulatory timelines:** The ZaZiBoNa initiative has measured and published the review timelines for the 333 dossiers/applications reviewed to date. This needs to be improved to include the monitoring, measuring and publication of the time to finalisation of ZaZiBoNa dossiers/applications in the individual participating countries.
• **Capacity building and training of assessors:** The ZaZiBoNa initiative has successfully facilitated and enabled the training of assessors in the 16 SADC countries. Going forward, the training and capacity-building activities should be separated from assessment activities, which will enable countries to second only competent assessors and inspectors, improving the effectiveness and efficiency of the initiative.
• **Information for applicants:** Requirements, guidelines, timelines and the process for submission of dossiers/applications to ZaZiBoNa should be made available on all participating country websites, including the contact details of the focal person.
• **Transparency of process and decision making:** Since 2017, the ZaZiBoNa initiative has prepared scientific summaries for approved medicinal products. These should be made available on the ZaZiBoNa and country websites.
• **Establishment of a regional medicines authority:** In the short-term, a regional unit hosted in one of the member countries to centrally receive ZaZiBoNa applications and coordinate communication with applicants should be piloted with the goal to establish a SADC regional medicines authority in the near future.

### Study Limitations

The scope of this study was limited to the ZaZiBoNa initiative’s process and operating model. In future, it would be helpful to obtain quantitative data to support these views which would include actual metrics of the time taken to register the medicinal products in the individual countries after a ZaZiBoNa recommendation. The status of commercialisation and pricing of the medicinal products in the individual countries as well as the factors influencing this could be the subject of a future study.

## Conclusion

This study identified the strengths of the ZaZiBoNa initiative as well as the opportunities for improvement. The recommendations should further strengthen this initiative, enabling it to fulfil one of its mandates, to ensure timely patient access to quality medicines in the SADC region. Although this was not the focus of this study, the SADC member states are encouraged to sign and ratify the African Medicines Agency (AMA) treaty, as that is considered the future of medicines regulation in Africa.

## Data Availability Statement

The raw data supporting the conclusions of this article will be made available by the authors, on request.

## Author Contributions

TS designed the study, collected, and analysed the data and wrote the first draft of the manuscript. GM interpreted the results and reviewed subsequent drafts of the manuscript. SW designed the study, interpreted the results, and reviewed subsequent drafts of the manuscript. SS designed the study, interpreted the results, and reviewed subsequent drafts of the manuscript. All authors contributed to the article and approved the submitted version.

## Conflict of Interest

The authors declare that the research was conducted in the absence of any commercial or financial relationships that could be construed as a potential conflict of interest.

## Publisher’s Note

All claims expressed in this article are solely those of the authors and do not necessarily represent those of their affiliated organizations, or those of the publisher, the editors and the reviewers. Any product that may be evaluated in this article, or claim that may be made by its manufacturer, is not guaranteed or endorsed by the publisher.

## Abbreviations

AMRH: African Medicines Regulatory Harmonisation Initiative
EAC: East African Community
PEER: Process, Effectiveness and Efficiency Rating
SADC: Southern African Development Community
SAPAM: Southern African Programme on Access to Medicines and Diagnostics
WHO: World Health Organization.
