# Supplementary material for: Regulatory Authority Evaluation of the Effectiveness and Efficiency of the ZaZiBoNa Collaborative Medicines Registration Initiative: The Way Forward
Source: Front Med (Lausanne). 2022 Apr 25;9:898743. doi: 10.3389/fmed.2022.898743 (PMC9082034; doi:10.3389/fmed.2022.898743)
Supplement: Supplementary file 1 [file Data_Sheet_1.PDF]

CONFIDENTIAL

ZAZIBONA COLLABORATIVE MEDICINES REGISTRATION INITIATIVE

PROCESS EFFECTIVENESS & EFFICIENCY RATING (PEER)

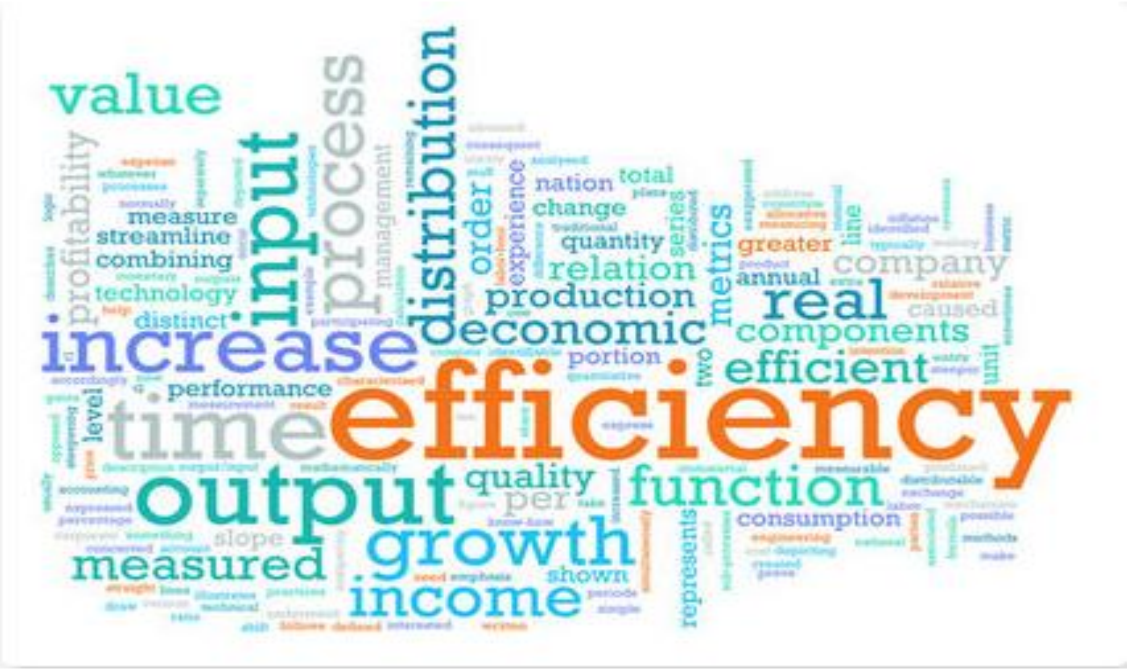

Pic taken from https://www.referenceforbusiness.com/management/De-Ele/Effectiveness-and-Efficiency.html

PEER QUESTIONNAIRE

July, 2021

Contacts:

Tariro Sithole
tariromakamure@gmail.com

Prof Stuart Walker
swalker@cirs.org

Prof Sam Salek
sssalek52@gmail.com

INTRODUCTION

The ZAZIBONA collaborative medicines registration initiative was established in 2013 and formally endorsed by the SADC health ministers in 2014. Since its inception, there has not been a formal and structured evaluation of this work sharing programme and for its future direction, although some feedback has been sought from manufactures through stakeholder meetings.

In recent years, there has been a drive within regulatory agencies to re-engineer their processes for improved efficiency and effectiveness and this often begins with a baseline evaluation of the current process to identify strengths and weaknesses. *Effectiveness* can be defined as 'doing the right thing' often measured by the value derived by customers/stakeholders from an organisation's processes or services while *Efficiency* can be defined as 'doing things right' which saves the organization time and resources.

Study Participants

The PEER Questionnaire is being sent to the active members of the ZaZiBoNa initiative namely, Botswana, Democratic Republic of Congo, Malawi, Mozambique, Namibia, South Africa, Tanzania, Zambia and Zimbabwe

AIM

The aim of this study is to evaluate the effectiveness and efficiency of the current operating model of the ZAZIBONA initiative including the challenges it faces as well as identifying opportunities for improvement.

STUDY OBJECTIVES

1. Obtaining the views of the individual authorities of the ZaZiBoNa initiative about the performance of the programme to date.
2. Identifying the challenges experienced by individual authorities throughout the life cycle of the ZaZiBoNa initiative.
3. Determining the strengths and weaknesses of the initiative
4. Identifying the ways of improving the performance of the working sharing programme.
5. Envisaging the strategy for moving forward

CONFIDENTIALITY

Thank you for agreeing to participate in this survey. **Your responses will be treated in strictest confidence and no identifiers of countries or respondents will be shared**

with any third party or made public. External reports or presentations of the data will include only blinded results together with appropriate analytical interpretations.

The questionnaire is divided into five short sections and will take 20 minutes to complete. Thank you for taking time to complete it. We value your input!

A. DEMOGRAPHICS

1. Please state the name of your country ________________________

2. Please provide your responses to the following questions by writing your answer in the space provided or ticking the relevant box.

   a. Age: _______years

   b. Sex: ☐ Male ☐ Female

   c. Number of years of regulatory experience: ______years

3. What is the total number of staff in your agency? _______

4. What is the number of reviewers of marketing authorization applications? ______

5. How many reviewers participate in the ZAZIBONA assessments? _______

6. Does your agency have a separate record of applications received for assessment under ZAZIBONA? ☐ Yes ☐ No

B. VIEWS ON THE BENEFITS OF THE ZAZIBONA INITIATIVE

Select your answers by ticking the relevant box(es)

1. In your view, what are 3 (or more) benefits of the ZAZIBONA initiative to date?

   ☐ Leadership commitment/Governance structure
   ☐ Clear Operating Model
   ☐ Shorter timelines for approval
   ☐ Information sharing among regulators
   ☐ Building of capacity for assessments
   ☐ Sustainable resource base because of self-funding by countries
   ☐ `Harmonisation of registration requirements across the region

☐ Other (Please specify) ______________________________________

2. What would you say are 3 (or more) <u>strengths of your process</u> for ZAZIBONA products <u>at country level</u> that other countries could learn from?

☐ Separate register and tracking of ZAZIBONA products
☐ Priority review of ZAZIBONA products
☐ Information on the submission process and timelines for ZAZIBONA products are available on your country website
☐ Products approved under ZAZIBONA are available on your country website
☐ Regular Committee meetings enabling timely finalisation of products after ZAZIBONA recommendation
☐ Other (Please specify) ______________________________________

3. How has the ZAZIBONA initiative benefited <u>member countries (regulators)</u>?

☐ Training to improve the performance of the assessors
☐ Provides the platform for interaction and information exchange with other regulators
☐ Shared workload resulting in shorter timelines for approval than in individual countries
☐ Enables application of high standards of assessment regardless of size of country or maturity of regulatory agency
☐ Improved quality of dossiers submitted
☐ Other (Please specify) ______________________________________

4. How has the ZAZIBONA initiative benefited <u>manufacturers (applicants)</u>?

☐ Reduced burden as they compile one dossier (modules 2 -5) for submission to multiple countries
☐ Savings on time and resources as they receive same list of questions from multiple countries enabling compilation of a single response package
☐ Shorter timelines for approval compared to that for the individual countries
☐ Access to various markets at the same time
☐ Other (Please specify) ______________________________________

5. How has the ZAZIBONA initiative benefited patients in your country or in the SADC region?

- ☐ Quicker access to quality assured medicines
- ☐ Reduced prices of medicines
- ☐ Increased availability of medicines
- ☐ Other (Please specify) ______________________________

C. VIEWS ON CHALLENGES OF THE ZAZIBONA INITIATIVE

Select your answers by ticking the relevant box(es)

1. In your view, what are 3 (or more) challenges of the ZAZIBONA initiative?

- ☐ Lack of detailed information on the process for applicants
- ☐ Low or decreasing number of applications for assessment
- ☐ Unequal workload among member countries
- ☐ Dependence on the countries' process for communication with applicants and expert Committees
- ☐ Lack of centralised submission and tracking
- ☐ Lack of jurisdiction power
- ☐ Other (please specify) ______________________________

2. In your view, what are 3 (or more) challenges that you face at country level in assessing/finalising ZAZIBONA products?

- ☐ Inadequate human resources
- ☐ Poor record keeping and tracking of ZAZIBONA products
- ☐ Lack of priority review for ZAZIBONA products
- ☐ ZAZIBONA work not recognized as part of agency work to be done during working hours
- ☐ Unpredictable schedule of Committee meetings
- ☐ Lack of buy-in from expert Committee(s)
- ☐ Failure by manufacturers to follow the requirement to submit the exact same dossier to all countries of interest
- ☐ Failure by manufacturers to adhere to deadlines for response to questions
- ☐ Other (Please specify) ______________________________

3. What are the challenges faced by <u>manufacturers</u> submitting applications to the ZAZIBONA initiative?

   - ☐ Differences in time to implementation of ZAZIBONA recommendations by member countries.
   - ☐ Lack of clarity about the process for submission and follow up in each country
   - ☐ Lack of information on country websites and the ZAZIBONA website about the process, milestones, timelines, pending and approved products
   - ☐ ZAZIBONA process is more stringent than some country processes
   - ☐ Differing labeling requirements in participating countries
   - ☐ Other (Please specify) ________________________________

D. <u>IMPROVING THE PERFORMANCE (EFFECTIVENESS AND EFFICIENCY) OF THE WORK SHARING PROGRAMME</u>

Select your answers by ticking the relevant box(es)

Effectiveness can be defined as 'doing the right thing' often measured by the value derived by customers/stakeholders from an organisation's processes or services while *Efficiency* can be defined as 'doing things right' which saves the organization time and resources.

1. What are 3 or more ways to improve the <u>effectiveness</u> of the ZAZIBONA initiative in your view?

   - ☐ Decision-making transparency e.g. publishing Public Assessment Reports
   - ☐ Make publicly available any information that might help applicants in managing their submissions - templates of documents, lists of Q&A, timelines and milestones, disclosure of internal SOPs, etc.
   - ☐ Consistency in application of guidelines and decisions
   - ☐ Use of risk-based approaches e.g. reliance pathways
   - ☐ Engagement and interaction with stakeholders
   - ☐ Publishing of pending products
   - ☐ Publishing of approved products
   - ☐ Minimise the need for country specific documents
   - ☐ Other (Please specify) ________________________________

2. What are 3 or more ways to improve the <u>efficiency</u> of the ZAZIBONA initiative in your view?

- ☐ Specific and clear requirements made easily available to applicants
- ☐ Compliance with target timelines by measuring and monitoring each milestone in the review process
- ☐ Use of robust IT systems
- ☐ Transparency on metrics and statistics e.g. % completed within timeline
- ☐ Improved central tracking of ZAZIBONA products
- ☐ Improved resources e.g. number of assessors
- ☐ Centralised system for submission of applications and communication with applicants
- ☐ Other (please specify) ____

E: ENVISAGING THE STRATEGY FOR MOVING FORWARD

1. Rate the following proposals to improve the current ZAZIBONA operating model from 1 – 3, number 1 representing what you think would be **most effective** in improving efficiency and number 3 the **least effective**. *Enter the appropriate number in the space provided before each proposal.*

☐ To continue with the current operating model unchanged.

☐ To continue with the current operating model but provide full information on the process including timelines and milestones as well as approved products on every participating country's website and on the ZAZIBONA website.

☐ The establishment of a regional administrative body to centrally receive and track ZAZIBONA applications which would be responsible for allocating work, apportioning the applicable fees to countries, tracking of applications and communication with applicants.

2. In your view, would the establishment of a SADC regional medicines agency, if legally possible, be the best strategy for improved performance going forward?

   ☐ Yes ☐ No

   Please explain why?____

   ____

3. In conclusion, what other strategies not previously highlighted can you think of that would strengthen the ZAZIBONA initiative going forward?

____

____

____

____

Please feel free to use the comment box below to elaborate on any of your answers or to highlight questions and answers that you believe should have been included in this questionnaire.

Name of person completing the questionnaire: ___________________________

Title (position): _________________________________

Date: ________________

Thank you for your time and help

Please do not copy, distribute or translate without the copyright holder's permission
